# Spino-pelvic radiological parameters in normal Indian population

**DOI:** 10.1051/sicotj/2016003

**Published:** 2018-04-30

**Authors:** Roop Singh, Sushil Kumar Yadav, Sushma Sood, Rohtas Kumar Yadav, Ravi Rohilla

**Affiliations:** 1 Department of Orthopaedic Surgery, Paraplegia & Rehabilitation, Pt. B.D. Sharma PGIMS Rohtak 124001 Haryana India; 2 Department of Physiology, Pt. B.D. Sharma PGIMS Rohtak 124001 Haryana India; 3 Department of Radiodiagnosis, Pt. B.D. Sharma PGIMS Rohtak 124001 Haryana India; 4 Department of Social and Preventive Medicine, Pt. B.D. Sharma PGIMS Rohtak 124001 Haryana India

**Keywords:** Spino-pelvic alignment, Radiologic parameters, Pelvic incidence, Lumbar lordosis angle

## Abstract

*Introduction*: There is increasing emphasis on the sagittal spino-pelvic alignment and its interpretation is of critical importance in the management of spinal disorders. A cross-sectional study of several spino-pelvic radiographic parameters was conducted to determine the physiological values of these parameters, to calculate the variations of these parameters according to epidemiological data, and to study the relationships among these parameters.

*Material and method*: Fifty normal healthy volunteers (29 males and 21 females) with no history of back pain were selected and were subjected to standing sagittal spino-pelvic radiographs. All the measurements of various radiographic parameters were performed with use of a software program. A statistical analysis was done to study the relationships among them.

*Results*: The mean values of pelvic incidence (PI) and lumbar Lordosis Angle (LLA) were 48.52 ± 8.99 and 58.78 ± 9.51, respectively. There was statistical difference between male and female parameters in LLA, lumbo-sacral angle (LSA), sacral horizontal angle (SHA), sacral inclination angle (SIA), sacropelvic angle (PRS1), pelvisacral angle (PSA), and PI. A majority of parameters had higher values for female subjects when compared to male subjects. PI was positively correlated with LLA, pelvic angle (PA), pelvic overhang (PO), pelvic tilt (PT), sacrofemoral distance (SFD), SHA, and sacropelvic translation (SPT), which were highly significant, whereas LLA was positively correlated with SHA and SIA only. PI and LLA were both negatively correlated with PSA, pelvic thickness (PTH), and PRS1.

*Conclusions*: This study presents the various spino-pelvic radiographic parameter values of a sample of the normal asymptomatic Indian population. There was significant difference in radiographic parameters between males and females in about half of the parameters studied in the sample. The values obtained are comparable with the values presented as normal in the literature. A comparison of the study results with data published about other populations revealed no differences in any of the pelvic parameters between the Indian, Brazilian, and Korean populations.

## Introduction

The sagittal spino-pelvic alignment pattern varies from one individual to another and is specific to each person. The vertebral column plays an important role in the support and locomotion of the human body. An understanding of the elements that compose it is essential for learning about its role in body balance and alignment. Many investigators have reported the importance of the sagittal plane contour in the normal function of the spine and in various diseased states [1, 2].

To analyze the consequences of changes in sagittal balance in each individual, we need to understand the normal parameters for the population. The judgment of normality can be made possible by analyzing the normal patterns of sagittal curvature and characteristics of each pattern of sagittal curvatures. If sagittal alignment is abnormal, more expenditure of energy and high demand on the dynamic and static stabilizer are required to compensate the abnormal sagittal alignment for balance [3, 4].

Several studies have evaluated the relationship between the position of the pelvis and spinal alignment [4–16]. However, it is important to know the values of these radiographic parameters in healthy individuals, without spinal disease. Although some studies address these parameters, it is interesting to evaluate them in a specific population as there are structural differences between different population groups. Various studies have been conducted with evaluations of individuals from the European, Causcasian, Brazilian, and Korean populations [1–3, 10–12, 17]. However, a similar study has not yet been conducted for the Indian population.

The objective of this study is to observe the parameters of sagittal and spino-pelvic balance in a sample of the Indian population consisting of volunteer asymptomatic individuals, in order to establish the relationship between these parameters, age, and sex and to compare the results with those of other studies that cover other population groups.

## Materials and methods

The present study was conducted from May 2012 to November 2014 in a tertiary care center. Fifty subjects agreed to participate in the study. All were volunteers and met the following inclusion criteria: an age between 18 and 50 years, no history of a spinal disorder or spinal surgery, and no radiographic abnormality detected prior to or during the study. Hip, knee, and ankle abnormalities were ruled out by clinical examination. All volunteers provided informed consent. The study population consisted of 50 volunteers (29 men and 21 women), with a mean age of 31.14 ± 9.62 years. The epidemiological and morphological characteristics of this cohort were obtained from the following data: age, gender, weight, and height. The body mass index was calculated as the weight in kilograms divided by the square of the height in meters.

Informed written consent was obtained from all the subjects participating in the study. The institutional review board cleared the study and ethical clearance was taken.

Each volunteer was thoroughly examined clinically to rule out any obvious spinal pathology and was subjected to sagittal spino-pelvic radiographs.

### Sagittal spino-pelvic radiographs

Lateral radiographs of the lumbo-pelvic region were taken using Philips digital radiography system. The participants were instructed to stand straight and relaxed, with their knees fully extended. The elbows were flexed, with both hands resting on a horizontal bar at the level of their shoulders. The film-to-focus distance was 2 m.

**Figure 1:**
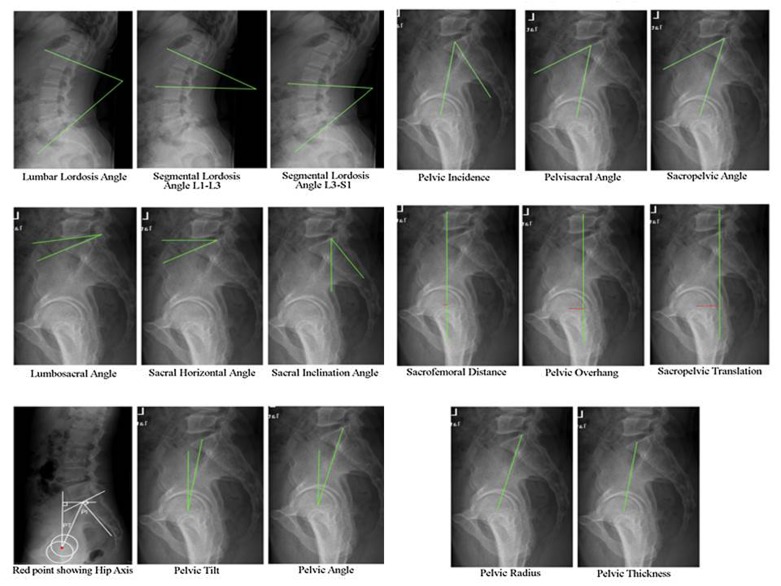
Shows schematic representation of various spino-pelvic parameter measurements on the radiographs.

The following angles (Figure 1) were measured on the sagittal spino-pelvic radiographs using open source software OsiriX (version 3.8.1, Pixmeo, Geneva, Switzerland) downloaded from http://www.osirix-viewer.com/.

- Lumbar Lordosis Angle (LLA) – The angle between the cephalad endplate of the first lumbar vertebra and the cephalad endplate of sacrum.
- Segmental Lordosis Angle (SLA) L1-L3 – The angle between the cephalad endplate of the first lumbar vertebra and the cephalad endplate of third lumbar vertebra.
- SLA L3-S1 – The angle between the cephalad endplate of the third lumbar vertebra and the cephalad endplate of sacrum.
- Lumbo-sacral angle (LSA) – The angle between the line along the upper border of sacrum and lower border of L5 vertebra.
- Sacral Horizontal Angle (SHA) – The angle between a horizontal line and a line drawn tangentially to the upper surface of sacrum.
- Sacral Inclination Angle (SIA) – The angle between the line along the posterior border of S1 body and the reference vertical line.
- Pelvic tilt (PT) – The angle between the line joining the hip axis (midpoint of bicoxofemoral axis) and the center of the S1 endplate and the reference vertical line.
- Pelvic angle (PA) – The angle between the line joining the hip axis and the posterior corner of the S1 endplate and the reference vertical line.
- Pelvic incidence (PI) – The angle between the line joining the hip axis and the center of S1 endplate and the line orthogonal to the S1 endplate.
- Pelvisacral Angle (PSA) – The angle between the line joining the hip axis and the center of S1 endplate and the line along the S1 endplate.
- Sacropelvic angle (PRS1) – The angle between the line joining the hip axis and the posterior corner of the S1 endplate and the line along the S1 endplate.
- Sacrofemoral distance (SFD) – The horizontal distance between the reference vertical line through the hip axis and the reference vertical line through the anterior corner of the S1 endplate.
- Pelvic overhang (PO) – The horizontal distance between the reference vertical line through the hip axis and the reference vertical line through the center of the S1 endplate.
- Sacropelvic translation (SPT) – The horizontal distance between the reference vertical line through the hip axis and the reference vertical line through the posterior corner of the S1 endplate.
- Pelvic Radius (PR) – The distance of the line joining the hip axis and the posterior corner of the S1 endplate.
- Pelvic thickness (PTH) – The distance of the line joining the hip axis and the center of S1 endplate.
- Lordosis Tilt Angle (LTA) – The angle between the anterior superior edge of S1 and the anterior superior edge of L1 with the reference vertical line is defined as the lordosis tilt angle. By convention, this angle is expressed as a negative value if the limit of the lumbar lordosis is posterior to the anterior aspect of S1, and positive if it is anterior to S1.

### Statistical analysis

Collected data were entered in the MS Excel spreadsheet, coded appropriately, and later cleaned for any possible errors in SPSS (Statistical Package for Social Studies) for Windows version 20. Categorical data were presented as percentage (%). Normally distributed data were presented as means and standard deviation, or 95% confidence intervals (CIs). For comparing two groups containing quantitative variables, independent sample *t*-test was used. In case of violation of normality, Mann-Whitney test was used. Pearson’s correlation was used for measuring correlation coefficient between two quantitative variables. In case of qualitative variables, Spearman’s correlation coefficient was applied. All tests were performed at a 5% level significance, thus a difference was significant if the value was less than 0.05 (*p* value < 0.05).

## Results

Table 1 shows the demographic data of study population. Of the 50 volunteers analyzed, 29 were males and 21 were females with a mean body mass index (BMI) of 24.72 ± 2.36 kg/m^2^.

**Table 1:** Demographic data study population.

	Mean ± *SD*, *n* = 50
Age (years)	31.14 ± 9.62
Sex	
Male	29
Female	21
Height (m)	1.64 ± 0.064
Weight (kg)	66.34 ± 5.33
BMI (kg/m^2^)	24.72 ± 2.36

Table 2 shows values of various radiological parameters of the study group. The mean values of PI and LLA were 48.52 ± 8.99 and 58.78 ± 9.51, respectively.

**Table 2:** Findings on sagittal spino-pelvic radiographs among study group.

Radiographic parameter	Mean ± *SD* (range) *n* = 50
Lumbar Lordosis Angle (LLA) (°)	58.78 ± 9.51 (37 to 79)
Lumbo-sacral angle (LSA) (°)	10.56 ± 3.58 (5 to 20)
Pelvic angle (PA) (°)	13.86 ± 6.76 (2 to 39)
Pelvic incidence (PI) (°)	48.52 ± 8.99 (33 to 69)
Pelvic overhang (PO) (mm)	18.22 ± 12.78 (−9 to 61)
Pelvic radius (PR) (mm)	128.90 ± 8.86 (111 to 157)
Pelvisacral Angle (PSA) (°)	41.36 ± 9.11 (21 to 57)
Pelvic tilt (PT) (°)	9.30 ± 7.16 (−5 to 37)
Pelvic thickness (PTH) (mm)	116.94 ± 9.41 (99 to 147)
Sacrofemoral distance (SFD) (mm)	4.52 ± 12.51 (−23 to 44)
Sacral Horizontal Angle (SHA) (°)	39.14 ± 7.05 (22 to 55)
Sacral Inclination Angle (SIA) (°)	48.62 ± 6.62 (35 to 62)
Segmental Lordosis Angle (SLA) L1-L3 (°)	17.54 ± 3.81 (9 to 25)
Segmental Lordosis Angle (SLA) L3-S1 (°)	43.46 ± 8.15 (28 to 64)
Sacropelvic angle (PRS1) (°)	37.02 ± 8.05 (18 to 52)
Sacropelvic translation (SPT) (mm)	30.82 ± 13.82 (4 to 75)
Lordosis Tilt Angle (LTA) (°)	−2.48 ± 4.98 (−16 to 10)

Table 3 shows the statistical comparison of various radiographic parameters according to gender. There was statistical difference between male and female parameters in LLA, LSA, SHA, SIA, PRS1, PSA, and PI. A majority of parameters had higher values for female subjects when compared to male subjects.

**Table 3:** Pelvic and spinal parameters according to sex.

Parameter	Gender	*N*	Mean	*SD*	*p* value
Height	M	29	1.6866	.03618	.001
	F	21	1.5762	.02889	
Weight	M	29	67.9655	4.30517	.010
	F	21	64.0952	5.88986	
BMI	M	29	23.9041	1.50582	.008
	F	21	25.8562	2.86638	
LLA	M	29	56.2069	9.54081	.023
	F	21	62.3333	8.46955	
SLA1	M	29	17.0000	4.01782	.243
	F	21	18.2857	3.46616	
SLA2	M	29	41.4828	6.40659	.059
	F	21	46.1905	9.59489	
LSA	M	29	9.3793	1.98950	.005
	F	21	12.1905	4.58933	
SHA	M	29	37.0000	6.26783	.010
	F	21	42.0952	7.16174	
SIA	M	29	46.7241	4.97060	.026
	F	21	51.2381	7.78399	
PT	M	29	9.1724	8.40097	.884
	F	21	9.4762	5.19249	
PA	M	29	13.8621	7.81813	.998
	F	21	13.8571	5.14087	
PRS1	M	29	38.9310	8.61449	.047
	F	21	34.3810	6.50750	
PSA	M	29	43.7241	9.32698	.030
	F	21	38.0952	7.91773	
PI	M	29	46.3103	9.28556	.040
	F	21	51.5714	7.79469	
SPT	M	29	31.4828	16.00362	.695
	F	21	29.9048	10.39666	
PO	M	29	17.8276	14.85422	.802
	F	21	18.7619	9.55460	
SFD	M	29	3.4828	14.48058	.497
	F	21	5.9524	9.29234	
PR	M	29	130.6207	8.75220	.107
	F	21	126.5238	8.65805	
PTH	M	29	119.0345	9.74856	.064
	F	21	114.0476	8.30347	
LTA	M	29	−2.97	4.64	0.424
	F	21	−1.81	5.45	

Table 4 shows Correlation of Pelvic Incidence and Lumbar Lordosis Angle with other radiographic parameters, age, gender, and BMI. PI was positively correlated with LLA, PA, PO, PT, SFD, SHA, and SPT, which were highly significant, whereas LLA was positively correlated with SHA and SIA only. PI and LLA were both negatively correlated with PSA, PTH, and PRS1.

**Table 4:** Correlation of pelvic incidence and lumbar lordosis angle with other radiographic parameters, age, gender, and BMI.

Correlation of PI with	*r* value (*p* value)	Correlation of LLA with	*r* value (*p* value)
	Study group		Study group
LLA	0.543 (0.000)	PI	0.543 (0.000)
LSA	−0.168 (0.245)	LSA	0.111 (0.443)
PA	0.574 (0.000)	PA	−0.188 (0.185)
PO	0.614 (0.000)	PO	−0.157 (0.267)
TR	−0.456 (0.000)	TR	−0.166 (0.247)
PSA	−0.993 (0.000)	PSA	−0.558 (0.000)
PT	0.642 (0.000)	PT	−0.127 (0.367)
PTH	−0.574 (0.000)	PTH	−0.345 (0.015)
SFD	0.684 (0.000)	SFD	−0.019 (0.864)
SHA	0.630 (0.000)	SHA	0.821 (0.000)
SIA	0.403 (0.004)	SIA	0.570 (0.000)
PRS1	−0.978 (0.000)	PRS1	−0.588 (0.000)
SPT	0.527 (0.000)	SPT	−0.219 (0.122)
LTA	0.465 (0.001)	LTA	0.216 (0.132)
Age	0.364 (0.009)	Age	0.013 (0.926)
BMI	0.301 (0.034)	BMI	0.357 (0.011)
Gender	−0.290 (0.041)	Gender	−0.293 (0.039)

Table 5 shows variations in radiographic parametersin overall and in males and females in different populations. The parameters among European population were comparatively higher than other studied populations.

**Table 5:** Comparison of different populations.

	Population	Brazilian [3] (*n* = 50)		European [1] (*n* = 300)		Korean [2] (*n* = 86)		Indian (*n* = 50)	
		Mean	*SD*	Mean	*SD*	Mean	*SD*	Mean	*SD*
PI	Total	48.7	9.6	54.7	10.6	47.8	9.5	48.5	9.0
	Male	49.1	6.4	53	10.6	48.8	7.3	46.31	9.28
	Female	48.3	9.6	56	10	46.1	9.5	51.57	7.79
SS	Total	38	8.4	41.2	8.5	36.3	8.6	39.1	7.0
	Male	38.2	6.9	41	8.5	37.3	7.1	37.00	6.26
	Female	37.8	8.4	43.2	8.4	34.4	8.6	42.09	7.16
PT	Total	12.15	6.2	13.2	6	11.5	5.4	9.3	7.2
	Male	12.1	6.2	13	6	11.4	5.4	9.17	8.40
	Female	12.2	5.3	13.6	6	11.6	5.1	9.47	5.19

Table 6 summarizes the findings of the studies done so far and the findings of the present study.

**Table 6:** Existing studies and reported values.

Study	*N*	Age (years)	PR (mm)	Description
Jackson et al. (1998) [5]	50	39.4 ± 9.5	135 ± 8.6	Normal
Jackson et al. (2003) [6]	75	39	136.8 ± 8.9	Normal
Present study	50	31.14 ± 9.62	128.9 ± 8.86	Normal
	29		130.62 ± 8.75	Normal males
	21		126.52 ± 8.65	Normal females
Study	*N*	Age (years)	PTH (mm)	Description

Duval-Beaupere et al. (1992) [7]	17	29.4 ± 11.0	120 ± 7.5	Normal
Rajnics et al. (2001) [8]	25	35.1 ± 3.0	155.5 ± 19.3	Normal females
	15	33.5 ± 2.9	133.1 ± 15.3	Normal males
Present study	50	31.14 ± 9.62	116.94 ± 9.41	Normal
	29		119.03 ± 9.74	Normal males
	21		114.04 ± 8.30	Normal females
Study	*N*	Age (years)	PSA (°)	Description

During et al. (1985) [9]	52		41.3 ± 10.0	Normal
Itio (1991) [10]	18	72	33.2 ± 13.2	Normal
Present study	50	31.14 ± 9.62	41.36 ± 9.11	Normal
	29		43.72 ± 9.32	Normal males
	21		38.10 ± 7.91	Normal females
Study	*N*	Age (years)	PI (°)	Description

Duval-Beaupere et al. (1992) [7]	17	29.4 ± 11.0	51.8 ± 9.4	Normal
Rajnics et al. (2001) [8]	15	33.5 ± 2.9	53.6 ± 8.9	Normal males
	15	35.1 ± 3.0	55.1 ± 8.4	Normal females
Vialle et al. (2005) [1]	300	35.4 ± 12.0	54.7 ± 10.6	Normal
	190		53.0 ± 10.6	Normal males
	110		56.0 ± 10.0	Normal females
Roussouly et al. (2005) [12]	160	27	51.9 ± 10.7	Normal
Boulay et al. (2006) [13]	149	30.8 ± 6.0	53.1 ± 9.0	Normal
Legaye (2007) [14]	145	40.7 ± 18.7	50.2 ± 10.6	Normal
Janssen et al. (2009) [15]	30	27	53 ± 10	Normal males
	30	26	50 ± 10	Normal females
Mac-Thiong et al. (2010) [16]	354	37.9 ± 14.7	52.7 ± 10.0	Normal males
	355	37.7 ± 13.9	52.4 ± 10.8	Normal females
Lee et al. (2011) [2]	86	28.19	47.8 ± 9.3	Normal
	54		48.8 ± 7.3	Normal males
	32		46.1 ± 9.5	Normal females
Pratali et al. (2014) [3]	50	34.85	48.7 ± 9.6	Normal
	25	32.3	49.1 ± 6.4	Normal males
	25	37.4	48.3 ± 9.6	Normal females
Present study	50	31.14 ± 9.62	48.52 ± 8.99	Normal
	29		46.31 ± 9.28	Normal males
	21		51.57 ± 7.79	Normal females
Study	*N*	Age (years)	PRS1 (°)	Description

Jackson et al. (1998) [5]	50	39.4 ± 9.5	31.2 ± 7.9	Normal
Jackson et al. (2000) [18]	20	46	31 ± 8.7	Normal
Jackson et al. (2003) [6]	75	39	30.9 ± 9.8	Normal
Legaye (2007) [14]	145	40.7 ± 18.7	35.2 ± 9.6	Normal
Present study	50	31.14 ± 9.62	37.02 ± 8.05	Normal
	29		38.93 ± 8.61	Normal males
	21		34.38 ± 6.50	Normal females
Study	*N*	Age (years)	PT (°)	Description

Legaye et al. (1998) [19]	28	24 ± 5.8	11.9 ± 6.6	Normal males
	21		10.3 ± 4.8	Normal females
Roussouly et al. (2005) [12]	160	27	11.99 ± 6.46	Normal
Vialle et al. (2005) [1]	300	35.4 ± 12.0	13.2 ± 6	Normal
	190		13 ± 6	Normal males
	110		13.6 ± 6	Normal females
Boulay et al. (2006) [13]	149	30.8 ± 6.0	11.96 ± 6.44	Normal
Janssen et al. (2009) [15]	30	27	12 ± 5.7	Normal males
	30	26	11 ± 6.8	Normal females
Mac-Thiong et al. (2010) [16]	354	37.9 ± 14.7	13.4 ± 6.7	Normal males
	355	37.7 ± 13.9	12.7 ± 7.0	Normal females
Lee et al. (2011) [2]	86	28.19	11.5 ± 5.3	Normal
	54		11.4 ± 5.4	Normal males
	32		11.6 ± 5.1	Normal females
Pratali et al. (2014) [3]	50	34.85	12.15 ± 6.2	Normal
	25	32.3	12.1 ± 6.2	Normal males
	25	37.4	12.2 ± 5.3	Normal females
Present study	50	31.14 ± 9.62	9.30 ± 7.16	Normal
	29		9.17 ± 8.40	Normal males
	21		9.47 ± 5.19	Normal females
Study	*N*	Age (years)	SHA (°)	Description

Legaye et al. (1998) [19]	28	24 ± 5.8	41.9 ± 8.7	Normal males
	21		38.2 ± 7.8	Normal females
Roussouly et al. (2005) [12]	160	27	39.9 ± 8.2	Normal
Vialle et al. (2005) [1]	300	35.4 ± 12.0	41.2 ± 8.5	Normal
	190		41 ± 8.5	Normal males
	110		43.2 ± 8.4	Normal females
Boulay et al. (2006) [13]	149	30.8 ± 6.0	41.18 ± 6.96	Normal
Janssen et al. (2009) [15]	30	27	41 ± 8.6	Normal males
	30	26	39 ± 7.6	Normal females
Mac-Thiong et al. (2010) [16]	354	37.9 ± 14.7	39.3 ± 8.0	Normal males
	355	37.7 ± 13.9	39.8 ± 7.9	Normal females
Lee et al. (2011) [2]	86	28.19	36.3 ± 7.8	Normal
	54		37.3 ± 7.1	Normal males
	32		34.4 ± 8.6	Normal females
Pratali et al. (2014) [3]	50	34.85	38 ± 8.4	Normal
	25	32.3	38.2 ± 6.9	Normal males
	25	37.4	37.8 ± 8.4	Normal females
Present study	50	31.14 ± 9.62	39.14 ± 7.05	Normal
	29		37.0 ± 6.26	Normal males
	21		42.10 ± 7.16	Normal females
Study	*N*	Age (years)	PO (mm)	Description

Legaye et al. (1998) [19]	28	24 ± 5.8	22.6 ± 12.5	Normal males
	21		19.2 ± 7.9	Normal females
Present study	50	31.14 ± 9.62	18.22 ± 12.78	Normal
	29		17.83 ± 14.85	Normal males
	21		18.76 ± 9.55	Normal females
Study	*N*	Age (years)	LLA (°)	Description
Legaye et al. (1998) [19]	28	24 ± 5.8	61.4 ± 10.2	Normal males
	21		58.1 ± 10.8	Normal females
Boulay et al. (2006) [13]	149	30.8 ± 6.0	66.36 ± 9.47	Normal
Present study	50	31.14 ± 9.62	58.78 ± 9.51	Normal
	29		56.21 ± 9.54	Normal males
	21		62.33 ± 8.46	Normal females
Study	*N*	Age (years)	SLA L1-L3 (°)	Description

Present study	50	31.14 ± 9.62	17.54 ± 3.81	Normal
	29		17.0 ± 4.01	Normal males
	21		18.28 ± 3.46	Normal females
Study	*N*	Age (years)	SLA L3-S1 (°)	Description

Present study	50	31.14 ± 9.62	43.46 ± 8.15	Normal
	29		41.48 ± 6.40	Normal males
	21		46.19 ± 9.59	Normal females
Study	*N*	Age (years)	LSA (°)	Description

Present study	50	31.14 ± 9.62	10.56 ± 3.58	Normal
	29		9.37 ± 1.98	Normal males
	21		12.1 ± 4.58	Normal females
Study	*N*	Age (years)	SIA (°)	Description

Present study	50	31.14 ± 9.62	48.62 ± 6.62	Normal
	29		46.72 ± 4.97	Normal males
	21		51.23 ± 7.78	Normal females
Study	*N*	Age (years)	LTA (°)	Description

Roussouly et al. (2005) [12]	160	27	−5.71 ± 4.59	Normal
Present study	50	31.14 ± 9.62	−2.48 ± 4.98	Normal
	29		−.2.96 ± 4.64	Normal males
	21		−1.80 ± 5.45	Normal females

## Discussion

The current study yields a physiological standard for several angular pelvic and spinal parameters that describe spinal balance, measured in a cohort of 50 asymptomatic adult volunteers of Indian resident population.

In the past three decades, increasing emphasis is being placed on quantitative evaluation of the parameters of sagittal spino-pelvic alignment as it is useful for clinical application and treatment of spino-pelvic pathologies. The harmony among spino-pelvic parameters is therefore of significant importance [4–19]. However, for us to correctly understand the effects of the loss of sagittal balance on the quality of life of each individual, we must know the normal values of the parameters used to evaluate sagittal and spinopelvic balance in the population.

A statistically significant difference was found between PI and gender in the present study with higher values of PI in females. Vialle et al. reported similar results with statistically significant differences between genders (*p* < 0.05), with higher PI values for females [1]. However, a number of studies reported no relationship between PI and gender [7, 8, 14, 15].

A positive correlation was found between PI and age in the study group (*r* = 0.36; *p* < 0.01) while Vialle et al. reported no relationship in normal adults [1]. A significant positive correlation was found between PI and BMI in the study group (*r* = 0.30, *p* = 0.03). Boulay et al. found similar significant correlation of *r* = 0.41 (*p* = 0.005) in normal adults [13].

A number of studies evaluated the relationship between PI and LLA, reporting significant correlation of *r* = 0.40–0.74 (*p* < 0.001) [8, 12, 14, 19]. In the present study, we found a significant positive correlation in the study group (*r* = 0.54) with *p* < 0.001.

Itoi reported a correlation of *r* = −0.211 (*p* = 0.035) between PSA and LLA [10]. Similar significant negative correlation was present in our study with *r* = −0.56 (*p* < 0.001). High correlation was reported between PI and sacropelvic angle (PRS1) with *r* = −0.95 (*p* < 0.001) [14]. In the present study, significant negative correlation was found between PI and PRS1 with *r* = −0.98 (*p* < 0.001).

Jackson et al. found significant negative correlation (*r* = −0.80 to −0.62, *p* < 0.001) between PRS1 and LLA [6, 18]. In the present study, we also found significant negative correlation with *r* = −0.59 (*p* < 0.001). Legaye reported significant positive correlation between PRS1 and PR (*r* = 0.38–0.73; *p* < 0.001) [14]. In the present study, we also found significant correlation between these two spino-pelvic parameters (*r* = 0.46; *p* = 0.001).

In the present study, a significant correlation was found between LLA and other spino-pelvic parameters i.e. SHA (*r* = 0.82; *p* < 0.001) and SIA (*r* = 0.57; *p* < 0.001). There was no significant correlation regarding SFD, PO, and SPT. Similar significant correlation was found between PI and other spinopelvic parameters i.e. PA (*r* = 0.57), SFD (*r* = 0.68), PO (*r* = 0.61), and SPT (*r* = 0.53).

The mean values of PI (48.52 ± 8.99, *n* = 50) in the present study in the control group were found similar to the data reported in the literature for Korean (47.8 ± 9.5, *n* = 86) and Brazilian (48.7 ± 9.6, *n* = 50) populations and somewhat different from data of European population (54.7 ± 10.6, *n* = 300) [1–3].

India is a country of mixed population. This study presents the results of an analysis of a small sample of healthy individuals. It can be noticed that the values obtained from the sample are within the values described as normal in the literature. In our study, there were differences in the radiographic parameters (LLA, LSA, SHA, SIA, PRS1, PSA and PI) when compared by sex of the individuals evaluated. A majority of parameters had higher values for female when compared to male subjects. When we compare the average values and standard deviations obtained in this study with those published in the literature for European, Brazilian, and Korean populations, we can see that there were no differences for any of the pelvic parameters between the Indian, Brazilian, and Korean populations, even when compared by sex. The values of pelvic incidence of the European population were higher than those of the Indian population sample. Both for the total sample and in the comparison by sex, the values of sacral slope of the European population were higher than those of the Indian population sample. Both for the total population and for the female group, the pelvic tilt values of the European population were similar to those obtained for the sample population studied. These data show the importance of studies in this format, aimed at adjusting the radiographic parameters for different populations [1–3]. Furthermore, the normative values derived from the present data can be utilized clinically to evaluate spinal deformities and in deformity corrective measures (surgical/conservative) targeting to achieve normal spino-pelvic balance in the Indian population.

## Conclusion

This study presents the various spino-pelvic radiographic parameter values of a sample of the normal asymptomatic Indian population. There was significant difference in radiographic parameters between males and females in about half of the parameters studied in the sample. The values obtained are comparable with the values presented as normal in the literature. A comparison of the study results with data published about other populations revealed no differences in any of the pelvic parameters between the Indian, Brazilian, and Korean populations. There were differences in pelvic incidence between the Indian and European populations both in the total sample and in the male and female groups. There were differences in sacral slope between the Indian and European populations in relation to the total sample and the female group. More extensive studies are needed to further validate the findings of the present study.

## Conflict of interest

The authors declare no conflict of interest.
